# Auditory brainstem response in infants and children with autism spectrum disorder: A meta‐analysis of wave V

**DOI:** 10.1002/aur.1886

**Published:** 2017-10-31

**Authors:** Oren Miron, Andrew L. Beam, Isaac S. Kohane

**Affiliations:** ^1^ Department of Biomedical Informatics Harvard Medical School Boston MA

**Keywords:** auditory, event related potential, biomarker, infants, children

## Abstract

Infants with autism spectrum disorder (ASD) were recently found to have prolonged auditory brainstem response (ABR); however, at older ages, findings are contradictory. We compared ABR differences between participants with ASD and controls with respect to age using a meta‐analysis. Data sources included MEDLINE, EMBASE, Web of Science, Google Scholar, HOLLIS, and ScienceDirect from their inception to June 2016. The 25 studies that were included had a total of 1349 participants (727 participants with ASD and 622 controls) and an age range of 0–40 years. Prolongation of the absolute latency of wave V in ASD had a significant negative correlation with age (R2 = 0.23; *P* = 0.01). The 22 studies below age 18 years showed a significantly prolonged wave V in ASD (Standard Mean Difference = 0.6 [95% CI, 0.5–0.8]; *P* < 0.001). The 3 studies above 18 years of age showed a significantly shorter wave V in ASD (SMD = −0.6 [95% CI, −1.0 to −0.2]; *P* = 0.004). Prolonged ABR was consistent in infants and children with ASD, suggesting it can serve as an ASD biomarker at infancy. As the ABR is routinely used to screen infants for hearing impairment, the opportunity for replication studies is extensive. ***Autism Res*** *2018, 11: 355–363*. © 2017 The Authors Autism Research published by International Society for Autism Research and Wiley Periodicals, Inc.

**Lay Summary:**

Our analysis of previous studies showed that infants and children with autism spectrum disorder (ASD) have a slower brain response to sound, while adults have a faster brain response to sound. This suggests that slower brain response in infants may predict ASD risk. Brain response to sound is routinely tested on newborns to screen hearing impairment, which has created large data sets to afford replication of these results.

The auditory brainstem response (ABR) is an auditory evoked potential that is recorded through electrodes on the scalp. The evoked potential is recorded as a waveform that is characterized by five waves, with the first wave (wave I) originating at the auditory nerve and the fifth wave (wave V) originating at the upper brainstem [Starr, 1976]. Recent publications show that wave V latency is prolonged in infants who were later diagnosed with autism spectrum disorder (ASD) [Cohen et al., 2013; Miron et al., 2015], a neurodevelopmental disorder that impairs social communication [American Psychiatric Association, 2013]. At older ages, some studies found prolonged wave V latency in ASD [Azouz, Kozou, Khalil, Abdou, & Sakr, 2014; Dabbous, 2012; Fujikawa‐Brooks, Isenberg, Osann, Spence, & Gage, 2010; Gillberg, Rosenhall, & Johansson, 1983; Kwon, Kim, Choe, Ko, & Park, 2007; Magliaro, Scheuer, Assumpção Júnior, & Matas, 2010; Ornitz, Mo, Olson, & Walter, 1980; Rosenblum et al., 1980; Rosenhall, Nordin, Brantberg, & Gillberg, 2003; Roth, Muchnik, Shabtai, Hildesheimer, & Henkin, 2012; Russo, Nicol, Trommer, Zecker, & Kraus, 2009; Sersen, Heaney, Clausen, Belser, & Rainbow, 1990; Skoff, Mirsky, & Turner, 1980; Sohmer & Student, 1978; Student & Sohmer, 1978; Tanguay, Edwards, Buchwald, Schwafel, & Allen, 1982; Tas et al., 2007; Tharpe et al., 2006; Ververi, Vargiami, Papadopoulou, Tryfonas, & Zafeiriou, 2015; Wong & Wong, 1991], while others found shorter wave V latency [Courchesne, Courchesne, Hicks, & Lincoln, 1985; Grillon, Courchesne, & Akshoomoff, 1989; Rumsey, Grimes, Pikus, Duara, & Ismond, 1984].

Prolonged wave V in infants with ASD may relate to brain overgrowth in infants with ASD [Courchesne et al., 2011; Courchesne, Carper, & Akshoomoff, 2003; Redcay & Courchesne, 2005], since head circumference correlates with ABR latency [Mitchell, Phillips, & Trune, 1989]. Brain overgrowth in infants with ASD often progressed to undergrowth by adulthood [Courchesne, Campbell, & Solso, 2011; Redcay & Courchesne, 2005]. This suggests that the finding of prolonged ABR in infants with ASD may progress to shorter ABR in adulthood.

Another finding that may relate to wave V prolongation in ASD is impaired myelination in ASD [Wolff et al., 2012], which may also relate to prolonged auditory cortical responses in ASD using magneto‐encephalography (MEG) [Gage, Siegel, Callen, & Roberts, 2003; Gage, et al. 2003; Oram Cardy, Flagg, Roberts, Brian, & Roberts, 2005; Oram Cardy et al., 2005; Roberts et al., 2013; Demopoulos et al., 2017; Demopoulos & Lewine, 2016; Edgar et al., 2015, 2014; Roberts et al., 2011, 2010]. Prolonged cortical response in children with ASD was also found in studies using Event‐Related Potential (ERP) of electroencephalogram (EEG), yet results are inconsistent across studies [Bruneau, Roux, Adrien, & Barthélémy, 1999; Courchesne, Kilman, Galambos, & Lincoln, 1984]. ASD abnormalities in MEG and EEG relate to cortical development more than ABR, which mainly reflects brainstem development. As the brainstem is mostly developed by infancy, ABR may prove especially useful in studying infants, particularly now with its widespread use in, and availability of data from newborn hearing screening [White, 2003]. If children with ASD have slower processing in the ABR it could effect the temporal synchrony in neural firing, which may explain the possible deficiency in temporal resolution and the audiovisual temporal processing in ASD [Stevenson et al., 2016, 2014].

Aside from wave V, other ABR waves also show prolongation in ASD [Rosenhall et al., 2003]. This meta‐analysis’ focus on Wave V is due to the relative consistency with which wave V appears in infant hearing screening [Driscoll & McPherson, 2010], thereby creating extensive opportunities for replication by others. If results from this meta‐analysis are born out by future studies, then wave V screening as part of the standard ABR for infant hearing screening could also be used for earlier detection of ASD. As the median age for ASD diagnosis is 4.1 years (Developmental Disabilities Monitoring Network Surveillance Year 2010 Principal Investigators & Centers for Disease Control and Prevention (CDC), 2014), earlier diagnosis could lead to earlier treatment, which may result in improved outcomes, even with existing treatment modalities [Dawson et al., 2010; Silverstein & Radesky, 2016; Siu et al., 2016].

## Methods and Materials

### Search

We performed a literature search of MEDLINE, EMBASE, Web of Science, Google Scholar, HOLLIS, and ScienceDirect from their inception to June 3rd 2016 (Fig. S1). The search yielded 50 studies on ABR in ASD compared to controls that were published between 1975 [Ornitz & Walter, 1975] and 2015 [Miron et al., 2015].

### Study Selection

**Figure 1:**
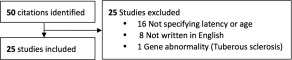
Inclusion of studies in meta‐analysis.

Studies were excluded if they (a) did not specify wave V latency or age values (b) were not written in English, or (c) used a sample comprised entirely of cases with a specific genetic abnormality (Fig. 1, Table S1).

After exclusion, 25 studies remained (50% inclusion proportion), with 1349 participants (727 participants with ASD and 622 controls), and an age range of 0–40 years.

### Data Extraction and Synthesis

Values for the analysis were extracted from the included articles and from data sent by authors (Table S2 and Figs. S2, S3, S4). Data extraction and analysis was performed three times to ensure accuracy. ASD prolongation percentage was calculated as mean wave V latency in the ASD group divided by mean wave V latency in the control group.

### Statistical Analysis

The analysis was performed with R statistical software (Version 3.3.1), with the packages “ggplot2” and “metafor”. The effect of confounding factors on ASD prolongation in wave V was examined using linear regression weighted by sample size. There was no significant correlation with male ratio, stimulus intensity (also known as loudness), stimulus rate, sweeps amount, and publication year (Table 1). The effect of polarity was examined with a *T*‐test and it did not have a significant effect (Table 1). A click stimulus was used in all of the studies in the meta‐analysis.

**Table 1:** Characteristics of the 25 Included Studies

Study (1st author)	Age (year)	Sample (N)	Male (%)	Intensity (dB nHL)	Rate (clicks/sec)	Sweeps (trials)	Publication (year)	Polarity
Cohen^a^	0.0	70	59	80	12.9	3,072	2013	Rarefaction
Miron^a^	0.1	60	80	85	39.1	2,000	2015	Alternating
Dabbous	2.4	50	66	90	27.5	N/A	2012	Rarefaction
Roth	2.6	52	83	85	39.1	2,000	2012	Alternating
Kwon	3.3	121	82	90	13	N/A	2007	N/A
Wong	3.4	129	72	80	10	2,048	1991	Rarefaction
Tas	3.9	42	75	80	16	2,000	2007	Alternating
Ververi	4.0	86	100	70	N/A	2,048	2015	N/A
Azouz	5.0	45	76	N/A	N/A	N/A	2014	N/A
Tanguay	5.5	28	86	72	20	1,500	1982	Rarefaction
Tharpe	5.7	36	90	80	21.1	2,000	2006	Alternating
Ornitz	6.0	15	N/A	68	10	1,024	1980	Rarefaction
Sohmer^b^	7.5	31	N/A	75	15	1,024	1978	N/A
Student^b^	8.0	24	N/A	75	10	N/A	1978	Alternating
Rosenhall (female)	9.6	54	0	80	22.5	1,012	2003	Alternating
Russo	9.8	39	73	N/A	13	3,000	2009	N/A
Rosenblum	9.8	12	67	60	10	1,000	1980	N/A
Skoff	10.3	36	53	60	10	2,000	1980	N/A
Rosenhall (male)	10.3	106	100	80	22.5	1,012	2003	Alternating
Fujikawa‐Brooks	10.8	40	88	75	19	1,024	2010	Alternating
Gillberg	11.3	55	65	80	N/A	2,048	1983	Alternating
Sersen	11.5	83	100	50	10.3	1,500	1990	Rarefaction
Magliaro	12.1	41	65	80	19	2,000	2010	Rarefaction
Rumsey	19.4	50	92	80	11	2,048	1984	Rarefaction
Courchesne^c^	19.6	28	86	70	37	2,750	1985	Rarefaction
Grillon^c^	21.7	16	100	70	7	2,000	1989	Rarefaction
**ASD prolongation effect**			**R^**2**^=0.006; NS**	**R^**2**^=0.0008; NS**	**R^**2**^=0.1; NS**	**R^**2**^=0.003; NS**	**R^**2**^=0.01; NS**	**T = 0.9; NS**

a Both studies used preterm infants in ASD and control groups.

b ASD participants overlap between both studies.

c ASD participants overlap between studies. N/A‐Not Available. NS‐Non significant weighted linear regression (p > 0.05), expect for Polarity which used a T‐test; dB nHL‐ Decibels above Normal Hearing Level.

Age is known to cause a decrease in the absolute latency of wave V and the Inter‐Peak Latencies (IPL) I–V. To examine if this decrease changes between ASD and controls, we examine the correlation of age with ASD prolongation for wave V and for IPL I–V using linear regression weighted by sample size. A similar analysis was performed for waves III and I (Fig. S5) and IPL I–III and III–V (Fig. S6). Comparison of Standard Mean Difference (SMD) of wave V in ASD vs. controls was performed to account for the different standard deviations in ASD and control for each study, which is not possible with percentage comparison. The SMD analysis made use of random effect model with the DerSimonian and Laird method [DerSimonian & Laird, 1986]. Analysis was done separately in studies with mean age below 18 years and mean age above 18 years.

## Results

### Wave V ASD Prolongation

**Figure 2:**
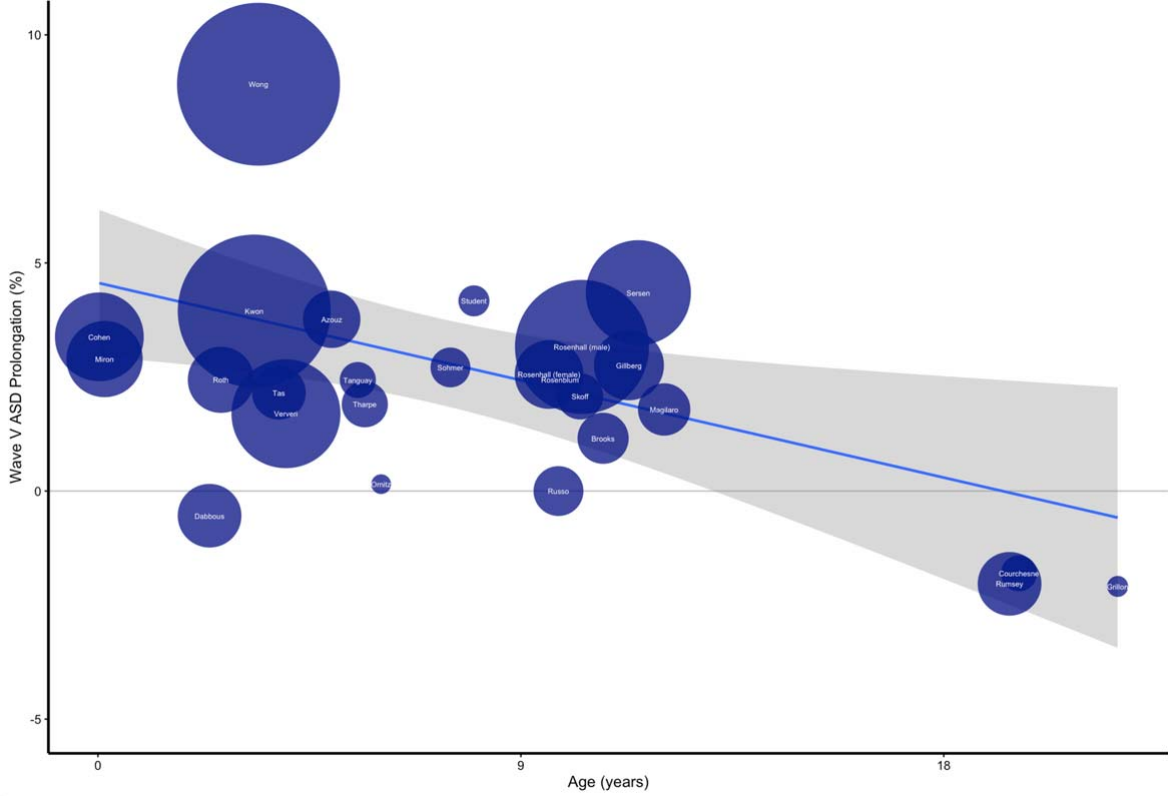
Correlation of wave V ASD prolongation and age. Legend: Y = Wave V ASD prolongation in percentage. X = mean age at time of ABR in years. Blue line = Linear regression. Grey area = Linear regression confidence interval of 95%. White names indicate first author name and circle size corresponds to sample size. For example, Circle “Cohen” represents Cohen et al. [2013] and the size corresponds to a sample size of 70 participants.

We first measured the effect of age on ASD prolongation of wave V using a weighted linear regression model. Age had a significant negative effect on prolongation of wave V in ASD (*R* ^2^ = 0.227, F_(1,24)_ = 7.04, *P* = 0.013, *B* = −0.23; Fig. 2).

### IPL I–V ASD Prolongation

**Figure 3:**
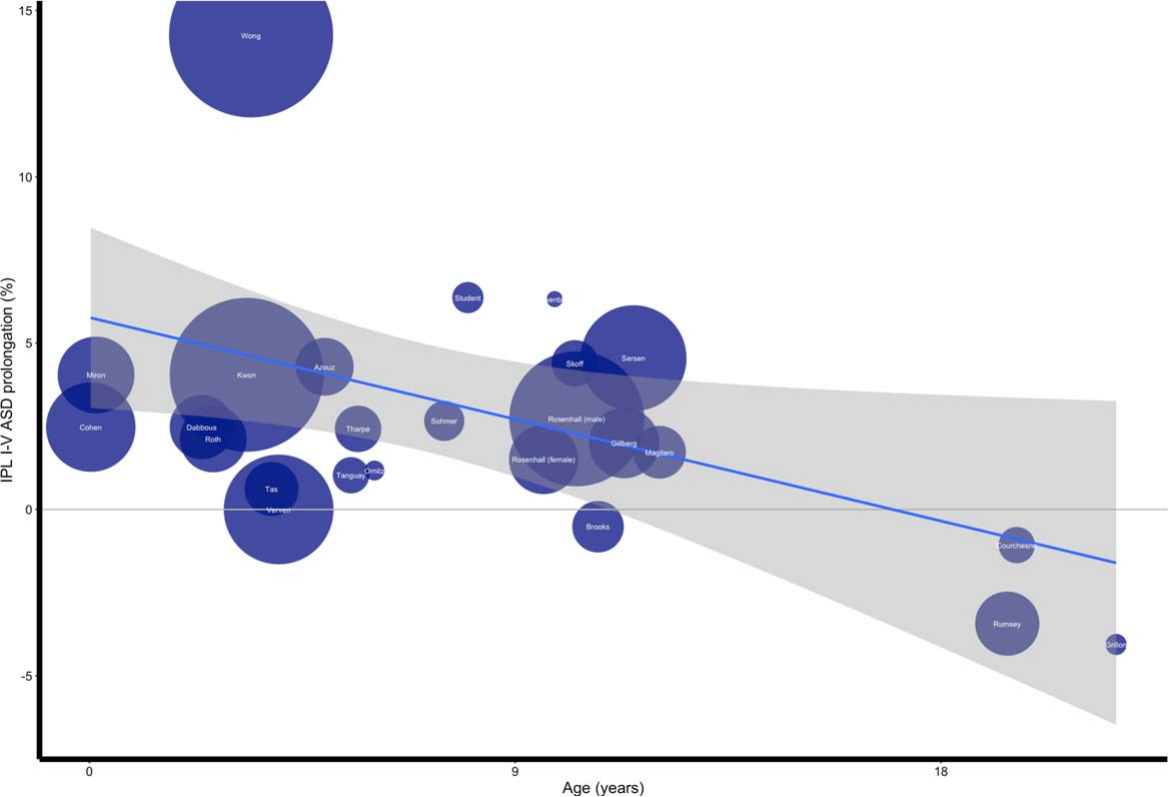
Correlation of IPL I–V ASD prolongation and age. Legend: Y = IPL I–V ASD prolongation in percentage. X = mean age at time of ABR in years. Blue line = Linear regression. Grey area = Linear regression confidence interval of 95%. White names indicate first author name and circle size corresponds to sample size. For example, Circle “Cohen” represents Cohen et al. [2013] and the size corresponds to a sample size of 70 participants.

To ensure that the effect of age on ASD prolongation of wave V did not stem from a low level problem or from arrangement of electrodes, we also analyzed the effect of age on ASD prolongation of IPL I–V using a weighted linear regression model (*R* ^2^ = 0.18, F_(1,23)_ = 5.07, *P* = 0.034, *B* = −0.34; Fig. 3).

### Standardized Mean Difference

**Figure 4:**
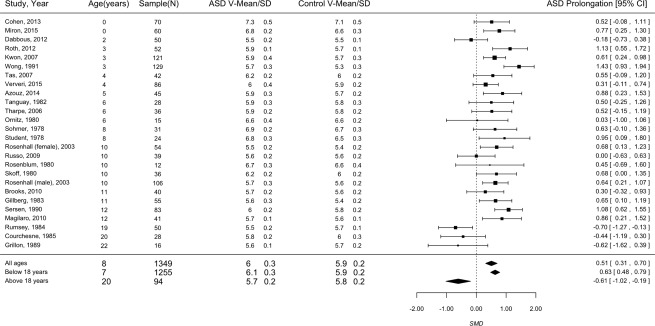
Forest plot of ASD prolongation. Legend: Study (first author), year (publication); Age (mean in years); Sample (combined for ASD and controls); V‐ Wave V latency; SD‐ Standard Deviation; Black square indicates SMD and error bars indicate confidence interval of 95%, which are indicated numerically under ASD Prolongation [95% CI]. Square size indicates the proportional weight of the study on the combined SMD.

In order to measure the SMD before and after adulthood, a separate analysis was performed for studies with mean age below 18 years of age and above 18 years of age. The 22 studies below 18 years of age showed significant ASD prolongation of wave V (SMD = 0.632, CI 0.476–0.788, *P* < 0.001). The three studies above 18 years of age showed significant ASD shortening of wave V (SMD = −0.607, CI −1.021 to −0.194, *P* = 0.004). When examining all 25 studies together, the ASD prolongation of wave V was significant (SMD = 0.505, CI 0.309–0.700, *P* < 0.001; Fig. 4).

## Discussion

This meta‐analytic study found that prolongation of absolute wave V latency in the ASD group compared to the control group has a significant negative correlation with age. Thus, the latency decrease with age that is known to occur in controls appears to happen more in ASD. Further, ABR studies below 18 years of age found wave V prolongation in ASD [Cohen et al., 2013; Dabbous, 2012; Kwon et al., 2007; Miron et al., 2015; Roth et al., 2012; Wong & Wong, 1991], while studies above 18 years of age found wave V shortening [Courchesne et al., 1985; Grillon et al., 1989; Rumsey et al., 1984]. ASD prolongation did not correlate significantly with study settings such as gender and stimulus intensity. The ASD prolongation of infants and children was significant in wave V, which originates from the highest area of the brainstem. In this regard, it is worth noting four studies that were excluded from the meta‐analysis for not specifying wave V but did specify IPL I–V (a higher part of wave V), where they found ASD prolongation in a combined sample of 755 children [Maziade et al., 2000; McClelland, Eyre, Watson, Calvert, & Sherrard, 1992; Taylor, Rosenblatt, & Linschoten, 1982; Thivierge, Bédard, Côté, & Maziade, 1990]. Another ABR component that was found abnormal in ASD is amplitude of wave I, which was higher than wave V in a greater percentage of ASD children compared to control children [Coutinho, Rocha, & Santos, 2002; Santos et al., 2017]. It will be important to examine ABR components in ASD in addition to absolute wave V latency, as wave V prolongation was also found in children with language delay, which highlights that the finding is not exclusive to ASD [Roth et al., 2012].

Results shown here demonstrated a negative effect of age on ASD prolongation at wave V, with prolongation in infancy and shortening at adulthood. This is similar to the negative effect of age on brain overgrowth in ASD, with overgrowth in infancy and undergrowth in adulthood [Courchesne et al., 2011; Redcay & Courchesne, 2005]. These studies suggest that in the first days of life, before the increased brain growth period, there may be a smaller brain size in ASD. Examining if this abnormality affects ABR requires that the post‐natal analysis for the first days of life should be done separately for subsequent pediatric development. Follow‐up studies to determine if the brain overgrowth and the V wave signal have any association also include: measuring the correlation between the head circumference (absolute size and growth rate) and V wave timing in both children with ASD and matched controls. Furthermore, given the impaired myelination observed in ASD [Wolff et al., 2012], the delayed V wave finding could be correlated with white fiber connectivity (i.e., with DTI MRI imaging). Such ASD studies on ABR and DTI could expend on ASD studies that used MEG and DTI to examine the connection of myelination and a delayed cortical response [Roberts et al., 2013, 2009]. Finally, the auditory sensitivity that burdens many individuals with ASD [Rosenhall, Nordin, Sandström, Ahlsen, & Gillberg, 1999] could also be correlated with the V wave delay, for example by impairing the temporal integration of auditory and visual information, which was shown to be abnormal in ASD [Stevenson et al., 2016; Stevenson et al., 2014].

ASD is a heterogeneous disorder with several subgroups [Doshi‐Velez, Ge, & Kohane, 2014; Karmel et al., 2010], which suggests that prolongation of wave V latency in ASD may occur in a subgroup of ASD cases. This notion was supported by several studies in the meta‐analysis [Miron et al., 2015; Rosenhall et al., 2003; Roth et al., 2012; Ververi et al., 2015]. For example, an infant study found wave V prolongation in 70% of ASD and 20% of controls [Miron et al., 2015]. The studies that were analyzed used different prolongation thresholds, which prevented a subgroup comparison in the meta‐analysis. However, this heterogeneity does suggest the opportunity in studying the genetics of the individuals identified with prolonged V wave. For example, assessing the genes involved in myelination and with variants previously associated with ASD. These include X‐linked dystrophin‐related protein 2 gene (DRP2) which regulates Schwann cell myelination and in which loss‐of‐function mutations have been found in patients with ASD [Toma et al., 2014], large non‐coding RNA mutations associated with delayed myelination and ASD [Talkowski et al., 2012], and mutations in ERBB4 associated with ASD and a tyrosine receptor kinase regulation of myelination [Gai et al., 2012]. However, rather than focusing on those specific genes, a genome‐wide approach is now feasible and affordable but it would require the reconsent and recontact of those families in which ASD and prolonged ABR V waves were found.

However, even without determining the specific mechanisms by which the prolonged V wave is associated with ASD, if it is found on replication studies to have sufficient specificity it could be used as a very low cost biomarker that fits into current pediatric practice. Therefore the only additional requirement would be the application of signal processing algorithms well within the capabilities of commodity consumer computing. Such a risk marker could refer infants at risk of ASD to more expensive tests and diagnostic evaluations, which cannot be performed on every newborn. Thus, the low cost and existing ABR test may help these tests in diagnosing ASD earlier and allow earlier and better treatment [Dawson et al., 2010].

## Supporting information

Additional Supporting Information may be found in the online version of this article at the publisher's website.

**Figure S1.** Search procedure
**Figure S2.** Extracting data from plot images
**Figure S3.** Funnel plot estimation of publication bias
**Figure S4.** Article comparison explanation
**Figure S5.** ASD prolongation in waves III and I
**Figure S6.** ASD prolongation in Inter‐Peak Latencies I–III and III–V
**Table S1.** Exclusion chart
**Table S2.** Contacting authors

Supporting Information
