# Supplementary material for: Auditory brainstem response in infants and children with autism spectrum disorder: A meta‐analysis of wave V
Source: Autism Res. 2017 Oct 31;11(2):355–63. doi: 10.1002/aur.1886 (PMC5836986; doi:10.1002/aur.1886)
Supplement: Supplementary file 1 — Figure S1. Search procedure Figure S2. Extracting data from plot images Figure S3. Funnel plot estimation of publication bias Figure S4. Article comparison explanation Figure S5. ASD prolongation in waves III and I Figure S6. ASD prolongation in Inter‐Peak Latencies I–III and III–V Table S1. Exclusion chart Table S2. Contacting authors [file AUR-11-355-s001.docx]

**Online Supplementary material**

Supplementary figure 1: Search procedure

The search was performed by the first author (OM), and was assisted by a librarian at Countway Library of Medicine (Boston, United States). The search utilized the software of the respective databases, and also made use of hand searching. The search terms used were:

MEDLINE

("Autistic Disorder"[Mesh] OR autis*[tiab](

AnD

("Evoked Potentials, Auditory, Brain Stem"[Mesh] OR ((auditory brainstem[tiab] OR auditory brain stem[tiab] OR auditory evoked[tiab]) AND (response*[tiab] OR potenial*[tiab])))

Embase

('autism'/exp OR autis*:ab,ti)

AND

('evoked brain stem auditory response'/exp OR ('auditory brainstem' NEAR/1 (response OR potential)):ab,ti OR ((brainstem OR 'brain stem') NEAR/1 'auditory evoked'):ab,ti)

Web of Science

TS="autis*"

AND

TS=(("auditory brainstem" NEAR/1 ("response" OR "potential")) OR (("brainstem" OR "brain stem") NEAR/1 "auditory evoked"))

Google Scholar, HOLLIS and ScienceDirect

“Auditory Brainstem Response Autism”

Supplementary Table 1: Exclusion chart

| Number | Study | Title | Reason for exclusion |
| --- | --- | --- | --- |
| 1 | Maziade et al 2000(1) | Prolongation of brainstem auditory-evoked responses in autistic probands and their unaffected relatives | No-V |
| 2 | Taylor et al 1982(2) | Auditory brainstem response abnormalities in autistic children | No-V |
| 3 | Mclelland et al 1992(3) | Auditory brainstem response screening for hearing loss in high risk neonates | No-V |
| 4 | Thivierge et al 1990(4) | Brain-stem auditory evoked response (BAER): Normative study in children and adults | No-V |
| 5 | Novick et al 1980(5) | An electrophysiologic indication of auditory processing defects in autism | No-V |
| 6 | Skoff et al 1986(6) | Brainstem auditory evoked potentials in autism | No-V |
| 7 | Gillberg et al 1987(7) | Neurobiological Findings In 20 Relatively Gifted Children With Kanner‐Type Autism Or Asperger Syndrome | No-V |
| 8 | Romero et al 2014(8) | AUDIOLOGIC AND ELECTROPHYSIOLOGIC EVALUATION IN THE AUTISTIC SPECTRUM DISORDER | No-V |
| 9 | Ho et al 1999(9) | Pervasive Developmental Delay in Children Presenting As Possible Hearing Loss | No-V |
| 10 | Demopoulos et al 2015(10) | Audiometric Profiles in Autism Spectrum Disorders: Does Subclinical Hearing Loss Impact Communication? | No-V |
| 11 | Kallstrand et al 2010(11) | Abnormal auditory forward masking pattern in the brainstem response of individuals with Asperger syndrome | No-V |
| 12 | Matas et al 2009(12) | Audiologic and electrophysiologic evaluation in children with psychiatric disorders | No-V |
| 13 | Fein et al 1981(13) | Clinical Correlates of Brainstem Dysfunction in Autistic Children | No-V |
| 14 | Steffenburg et al 1991(14) | Neuropsychiatric assessment of children with autism: a population-based study. | No-V |
| 15 | Yan Hua et al 2012(15) | Auditory abnormalities in children with autism.(Report) | No-V |
| 16 | Al-Ayadhi(16) | Auditory brainstem evoked response in autistic children in central Saudi Arabia | No-age |
| 17 | Zhang et al 2008(17) | Observation of the Brainstem Auditory Evoked Potentials in Autism Childhood | No-English |
| 18 | Zhang et al 2010(18) | Discussion on early diagnosis of brainstem auditory evoked potentials in Childhood Autism | No-English |
| 19 | Garruae et al 1984(19) | Auditory brain-stem evoked responses in normal and autistic children | No-English |
| 20 | Germano et al 2006(20) | Neurobiology of autism: Study of a sample of autistic children | No-English |
| 21 | Wu et al 2014(21) | The clinical analysis of results of brainstem auditory evoked potentials in 27 autism children | No-English |
| 22 | Wang et al 2009(22) | Auditory brainstem response and DPOAE in autism children | No-English |
| 23 | Wang et al 2010(23) | The Hearing Status and the Functions of Efferent System in Autistic Children | No-English |
| 24 | Rongqin Wu et al 2011(24) | The clinical applications of auditory brainstem response in juvenile with schizophrenia and autistism | No-English |
| 25 | Seri et al 1991(25) | Autism in tuberous sclerosis: evoked potential evidence for a deficit in auditory sensory processing | Tuberous sclerosis |

Supplementary Table 2: Contacting authors

An attempt was made to reach authors of studies that did not specify wave V latency or age, which is needed for the comparison between the studies (authors whose article provided sufficient data were not contacted). Of the authors that were contacted, the authors of three articles that published in 2014(26), 2013(27) and 2009(28) responded with the data (see table). The authors of older publications that were contacted did not have the data or did not respond. The author of a poster presentation from 1986(6) was contacted for more information and he replied that it was not saved, so only journal articles were used in the final analysis. An attempt was made to contact authors who did not publish in English to ask for an English version but in the 3 attempts that were made, no response was reached (18, 20, 29), so no further attempts were made.

| Corresponding Author | Response |
| --- | --- |
| Dr. Rania Abdou(26) | Sent means |
| Dr. Ira Cohen(27) | Sent means and gender ratio |
| Dr. Nina Kraus(28) | Sent means |
| Dr. Haim Sohmer(30) | Confirmed that his article and Student et al 1978(31) probably used the same children with ASD |
| Dr. Novick(5) | Attempting to find data |
| Dr. Maziade(1) | Data not saved |
| Dr. Thivierge(4) | Data not saved |
| Dr. Taylor(2) | Data not saved |
| Dr. McClelland(3) | Data not saved |
| Dr. Skoff(6) | Data not saved |
| Dr. Matas(12) | No reply |
| Dr. Al-Ayadhi(16) | No reply |
| Dr. Zhang(18) | No reply |
| Dr. Gerraue(19) | No reply |
| Dr. Germano(20) | No reply |
| Dr. Frizzo(8) | No reply |
| Dr. Fein(13) | No reply |

Supplementary Figure 2: Extracting data from plot images

For 3 articles that specified mean latencies only in plot images, the values were extracted from the image (Web-Plot-Digitizer <http://arohatgi.info/WebPlotDigitizer/>). Extracted values were later rechecked manually. Red dots indicate the extracted values of means and standard deviations.

Rosenhall et al 2003(32)


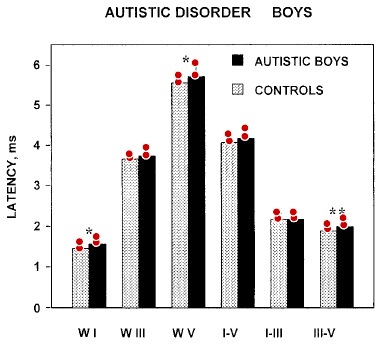

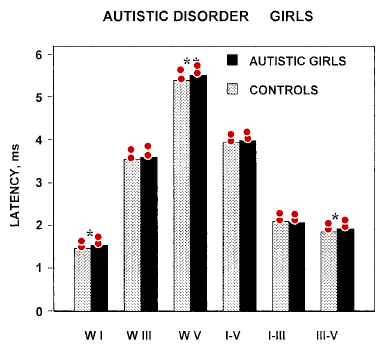


Tanguay et al 1982(33)


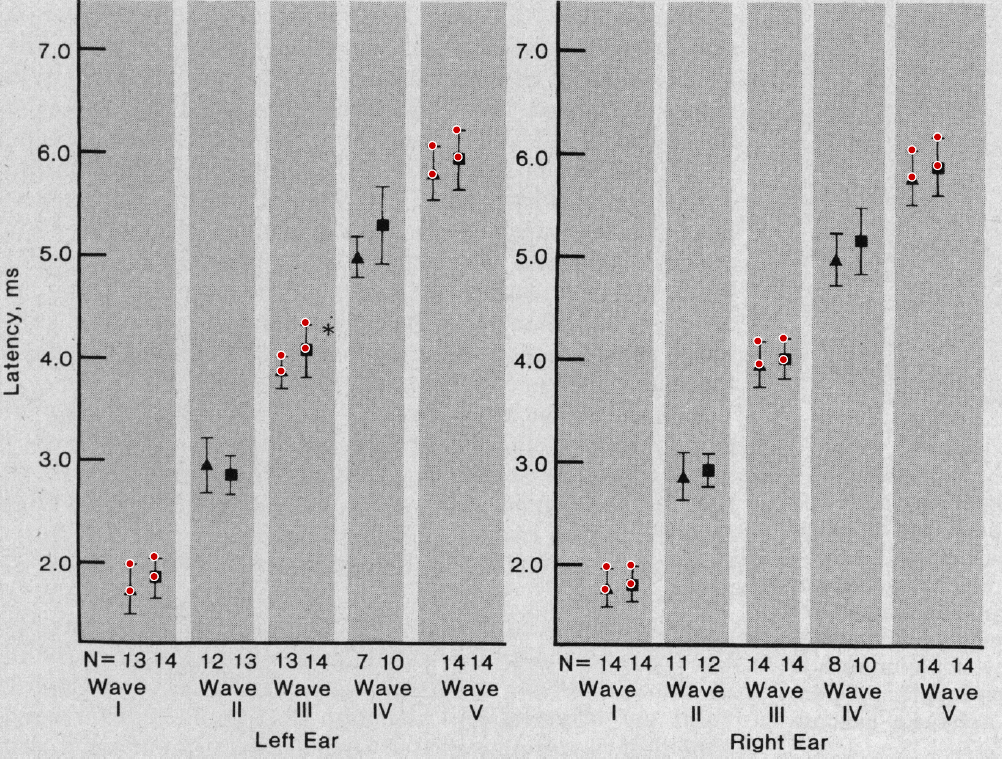


Courchesne et al 1985(34)


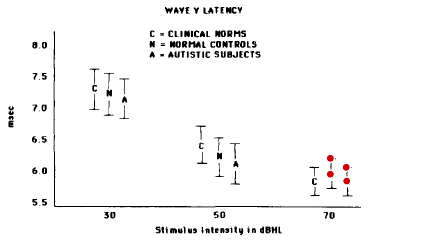


Supplementary Figure 3: Funnel plot estimation of publication bias

Legend: Y=Standard error of wave V ASD prolongation. X=Standardized Mean Difference of wave V ASD prolongation.

Supplementary Figure 4: Article comparison explanation

When articles used several sounds intensities, the analysis included the values from the intensities that were closest to the intensities of the other articles.(33, 34) One article specified all the waves of a certain intensity and only one wave for other intensities, which lead to the inclusion of the more detailed intensity.(40) Most articles specified latency for both ears, so in the few cases specified for left and/or right, the latencies were averaged. In one study a subsample of toddlers was compared to clinical norms based on young adults, so this subsample was not include in the analysis to avoid bias by the large age difference.(41) In studies using both an ASD group and mild/sub-clinical ASD group, the ASD group was included.(42, 43) Average age was calculated by averaging the control average age and ASD average age. In the few studies where average was not specified but range was, the minimum age and the maximum age were averaged. (30, 31, 44) Similarly, in the few studies where number of sweeps or rate was given as minimum and maximum values, the minimum and the maximum values were averaged.(30, 32, 34, 45) Comparison of hearing threshold exclusion was not possible due to studies using different thresholds. The majority of studies excluded abnormal hearing cases.(26, 27, 32–34, 40–43, 46–56) In cases where some latencies were not specified but could be calculated by the other latencies that were specified, such calculation was performed. For example, when IPL I-V was not specified but the absolute latencies of waves V and I were specified, the absolute latencies were used to calculate the IPL.

Supplementary Figure 5: ASD prolongation in waves III and I


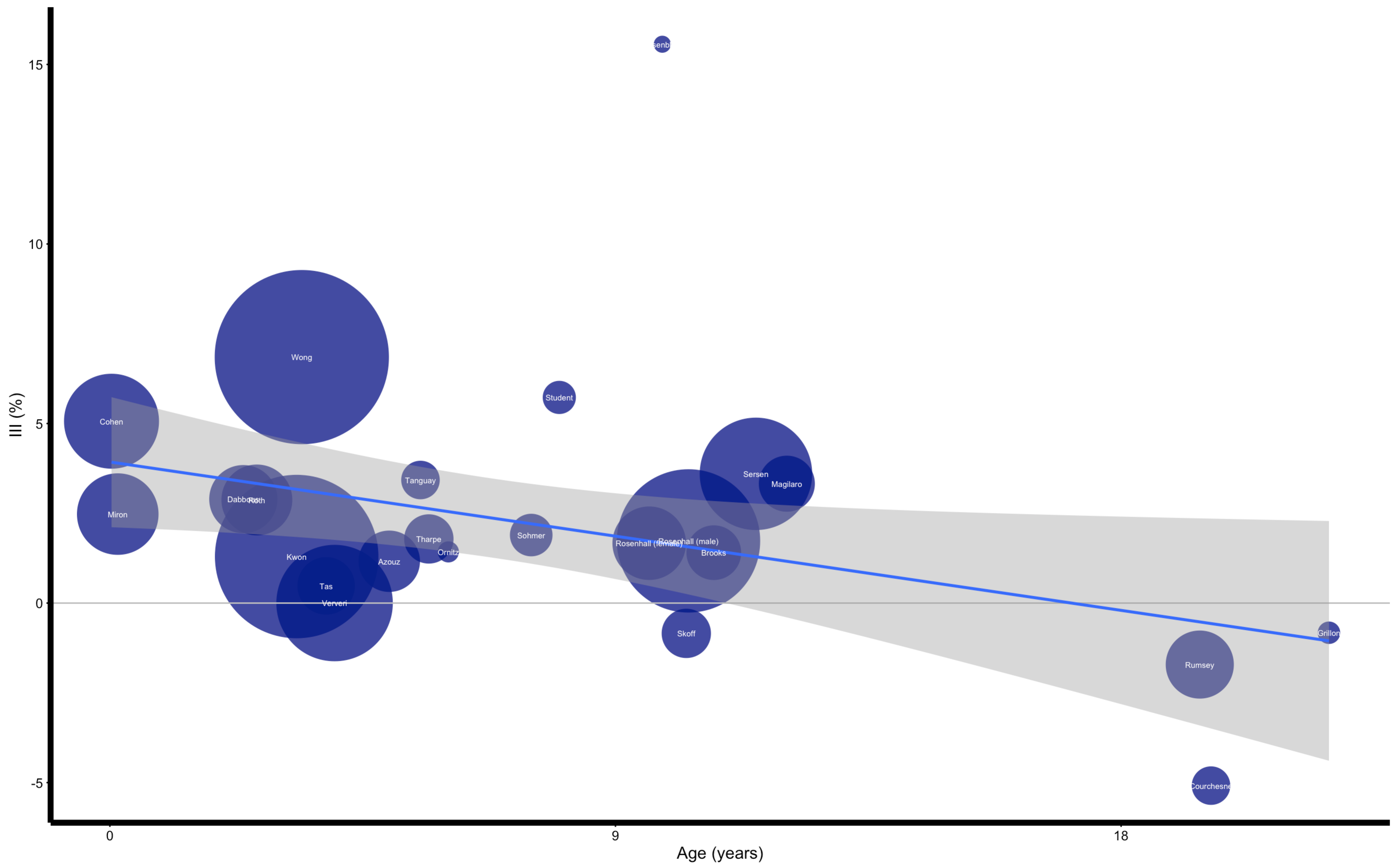

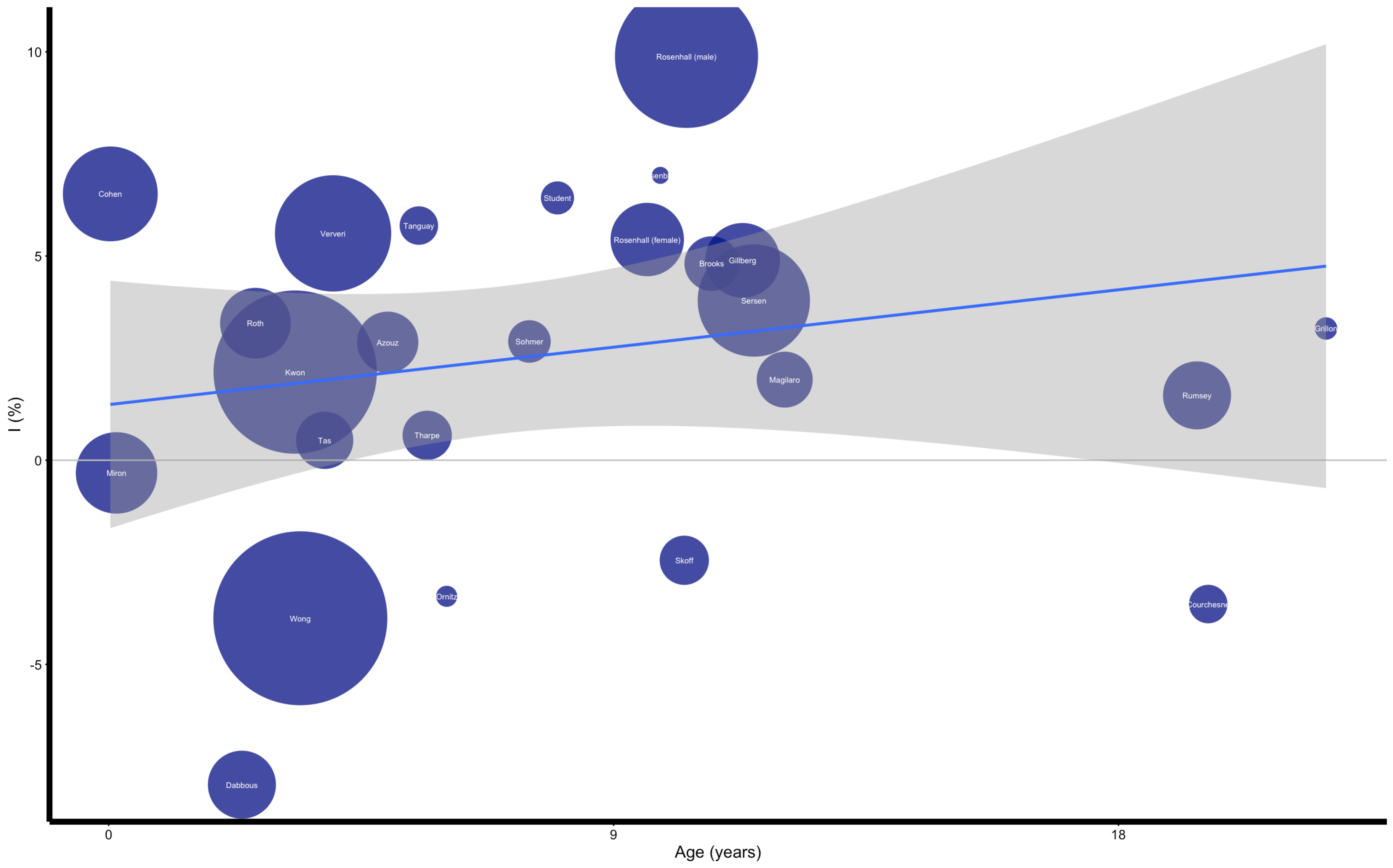
Legend: Y=Wave prolongation in percentage (III top & I bottom). X=mean age at time of ABR in years. Blue line=Linear regression. Grey area=Linear regression confidence interval of 95%. White names indicate first author name and circle size corresponds to sample size. For example, Circle “Cohen” represents Cohen et al 2013 and the size corresponds to a sample size of 70 participants. Wave III originates from a higher area compared to wave I.

Supplementary Figure 6: ASD prolongation in Inter-Peak Latencies I-III and III-V
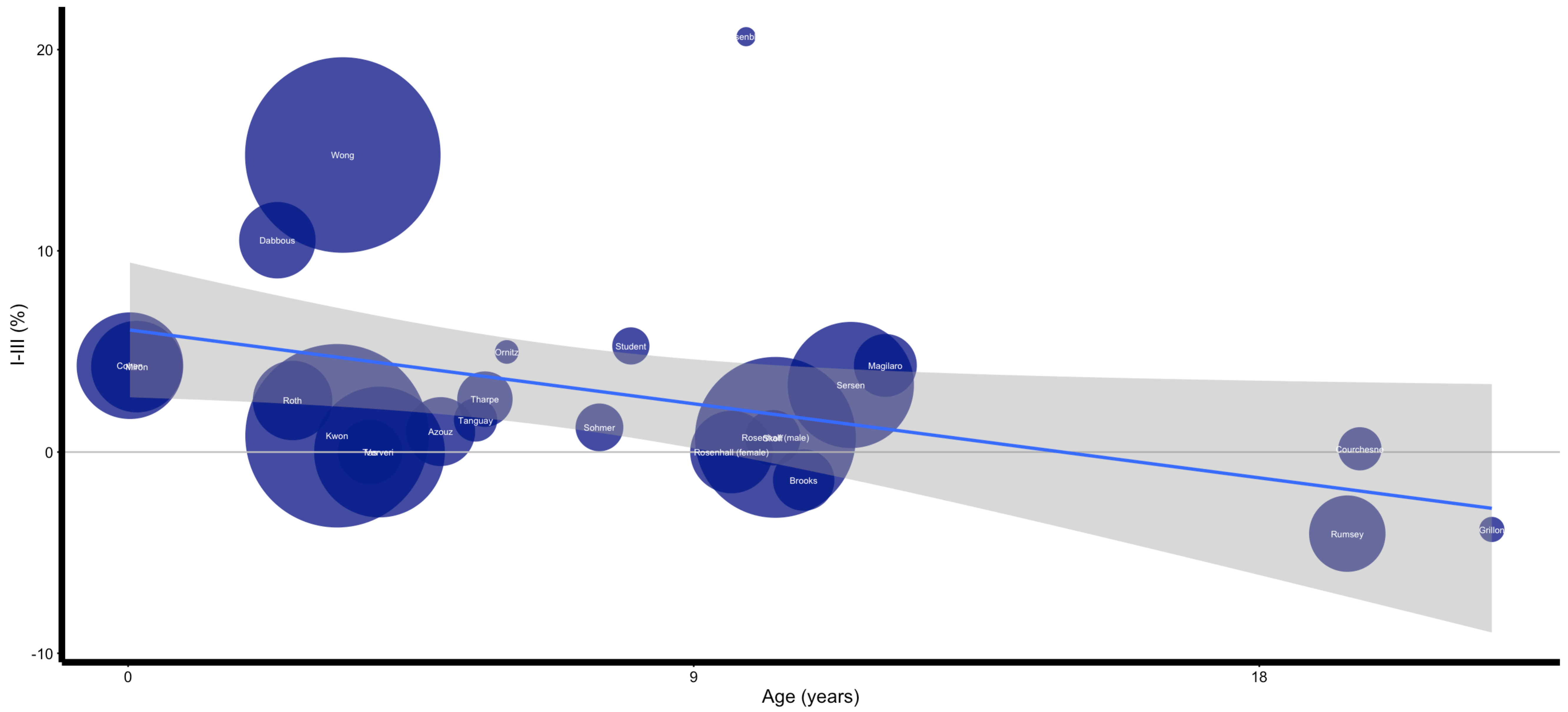


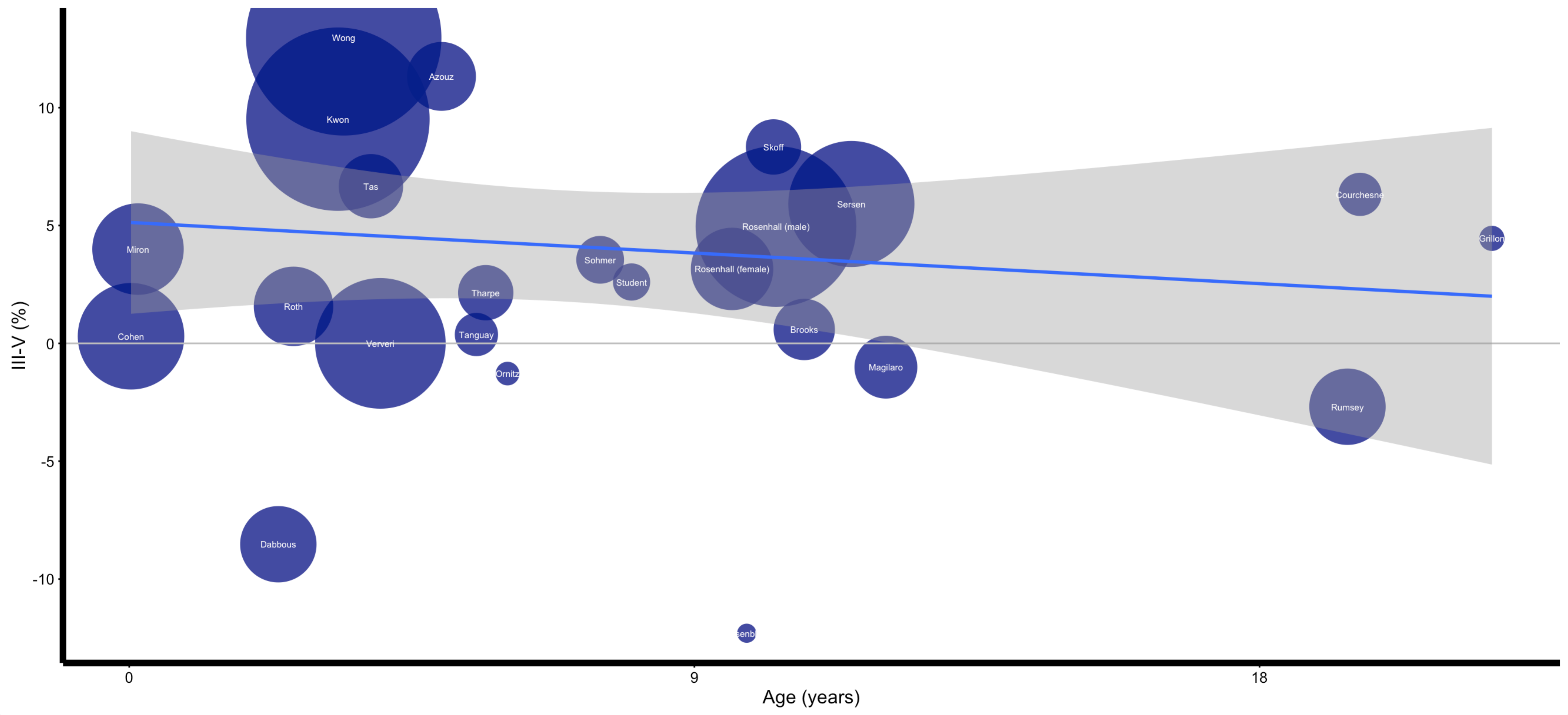
Legend: Y=Wave prolongation in percentage (I-III top & III-V bottom). X=mean age at time of ABR in years. Blue line=Linear regression. Grey area=Linear regression confidence interval of 95%. White names indicate first author name and circle size corresponds to sample size. For example, Circle “Cohen” represents Cohen et al 2013 and the size corresponds to a sample size of 70 participants. Inter-Peak latencies are measured as the latency difference between the absolute latency of wave I, III & V.

**SUPPLEMENTAL REFERENCES**

1. Maziade M, Mérette C, Cayer M, Roy MA, Szatmari P, Côté R, *et al. (2000): Prolongation of brainstem auditory-evoked responses in autistic probands and their unaffected relatives. Arch Gen Psychiatry*. 57(11): 1077–83.

2. Taylor MJ, Rosenblatt B, Linschoten L (1982): Auditory brainstem response abnormalities in autistic children. *Can J Neurol Sci*. 9(4): 429–33.

3. McClelland RJ, Eyre DG, Watson D, Calvert GJ, Sherrard E (1992): Central conduction time in childhood autism. *Br J Psychiatry*. 160: 659–63.

4. Thivierge J, Bédard C, Côté R, Maziade M (1990): Brainstem auditory evoked response and subcortical abnormalities in autism. *Am J Psychiatry*. 147(12): 1609–13.

5. Novick B, Vaughan HG, Kurtzberg D, Simson R (1980): An electrophysiologic indication of auditory processing defects in autism. *Psychiatry Res*. 3(1): 107–14.

6. Skoff BD, Fein D, McNally B, Lucci D, Humes-Bartlo M, Waterhouse L (1986): Brainstem auditory evoked potentials in autism. *Psychophysiology*

7. Gillberg C, Steffenburg S, Jakobsson G (1987): Neurobiological findings in 20 relatively gifted children with kanner-type autism or asperger syndrome. *Dev Med Child Neurol*. 29(5): 641–49.

8. Romero A, Gução A, Delecrode C, Cardoso A, Misquiatti A, Frizzo A (2014): Audiologic and electrophysiologic evaluation in the autistic spectrum disorder. *Rev. CEFAC*. 16(3): 707–14.

9. Ho PT, Keller JL, Berg AL, Cargan AL, Haddad J (1999): Pervasive developmental delay in children presenting as possible hearing loss. *Laryngoscope*. 109(1): 129–35.

10. Demopoulos C, Lewine JD (2016): Audiometric profiles in autism spectrum disorders: does subclinical hearing loss impact communication? *Autism Res*. 9(1): 107–20.

11. Källstrand J, Olsson O, Nehlstedt SF, Sköld ML, Nielzén S (2010): Abnormal auditory forward masking pattern in the brainstem response of individuals with asperger syndrome. *Neuropsychiatr Dis Treat*. 6: 289–96.

12. Matas CG, Gonçalves IC, Magliaro FC (2009): Audiologic and electrophysiologic evaluation in children with psychiatric disorders. *Braz J Otorhinolaryngol*. 75(1): 130–38.

13. Fein D, Skoff B, Mirsky AF (1981): Clinical correlates of brainstem dysfunction in autistic children. *J Autism Dev Disord*. 11(3): 303–15.

14. Steffenburg S (1991): Neuropsychiatric assessment of children with autism: a population-based study. *Dev Med Child Neurol*. 33(6): 495–511.

15. Hua TY, Yan XC, Ping JS, Xin SB, Bo WL, Lin W (2012): Auditory abnormalities in children with autism.

16. Al-Ayadhi LY (2008): Auditory brainstem evoked response in autistic children in central saudi arabia. *Neurosciences (Riyadh)*. 13(2): 192–93.

17. Zhang Nan, Li Yuru (2008): Observation of the brainstem auditory evoked potentials in autism childhood.

18. Zhang Y, Shen H, He D (2010): Discussion on early diagnosis of brainstem auditory evoked potentials in childhood autism. *National Medical Frontiers of China*. 5(2):

19. Garreau B, Tanguay P, Roux S, Lelord G (1984): [brain stem auditory evoked potentials in the normal and autistic child]. *Rev Electroencephalogr Neurophysiol Clin*. 14(1): 25–31.

20. Germanò E, Gagliano A, Magazù A, Calarese T, Calabrò ME, Bonsignore M, *et al. (2006): [neurobiology of autism: study of a sample of autistic children]. Minerva Pediatr*. 58(2): 109–20.

21. WU Xiao-qing, Chang He, WU Min, YAN Dong － mei (2014): The clinical analysis on results of brainstem auditory evoked potentials in 27 autism children. *Proceeding of Clinical Medicine*

22. Wang Sufang, Dong Xuelei, Wang Yongsheng (2009): Auditory brainstem response and dpoae in autism children.

23. Wang Chenrong, Hua Qingquan, Huang Zhiwu, Li Dan (2010): The hearing status and the functions of efferent system in autistic children.

24. Rongqin Wu, Shaojin Zhang, Guangqi Zhang, Chong Chen (2011): The clinical applications of auditory brainstem response in juvenile with schizophrenia and autistism.

25. Seri S, Cerquiglini A, Pisani F, Curatolo P (1999): Autism in tuberous sclerosis: evoked potential evidence for a deficit in auditory sensory processing. *Clin Neurophysiol*. 110(10): 1825–30.

26. Azouz HG, Kozou H, Khalil M, Abdou RM, Sakr M (2014): The correlation between central auditory processing in autistic children and their language processing abilities. *Int J Pediatr Otorhinolaryngol*. 78(12): 2297–2300.

27. Cohen IL, Gardner JM, Karmel BZ, Phan HT, Kittler P, Gomez TR, *et al. (2013): Neonatal brainstem function and 4-month arousal-modulated attention are jointly associated with autism. Autism Res*. 6(1): 11–22.

28. Russo N, Nicol T, Trommer B, Zecker S, Kraus N (2009): Brainstem transcription of speech is disrupted in children with autism spectrum disorders. *Dev Sci*. 12(4): 557–67.

29. Garreau B, Barthelemy C, Martineau J, Bruneau N, Lelord G (1985): [electrophysiologic aspects of infantile autism]. *Encephale*. 11(4): 145–55.

30. Sohmer H, Student M (1978): Auditory nerve and brain-stem evoked responses in normal, autistic, minimal brain dysfunction and psychomotor retarded children. *Electroencephalogr Clin Neurophysiol*. 44(3): 380–88.

31. Student M, Sohmer H (1978): Evidence from auditory nerve and brainstem evoked responses for an organic brain lesion in children with autistic traits. *J Autism Child Schizophr*. 8(1): 13–20.

32. Rosenhall U, Nordin V, Brantberg K, Gillberg C (2003): Autism and auditory brain stem responses. *Ear Hear*. 24(3): 206–14.

33. Tanguay PE, Edwards RM, Buchwald J, Schwafel J, Allen V (1982): Auditory brainstem evoked responses in autistic children. *Arch Gen Psychiatry*. 39(2): 174–80.

34. Courchesne E, Courchesne RY, Hicks G, Lincoln AJ (1985): Functioning of the brain-stem auditory pathway in non-retarded autistic individuals. *Electroencephalogr Clin Neurophysiol*. 61(6): 491–501.

35. Screening Data |Early Hearing Detection and Intervention (EHDI) | NCBDDD | CDC. https://ehdidash.cdc.gov/IAS/dataviews/view?viewId=26

36. EHDI Programs | Hearing Loss | NCBDDD | CDC. http://www.cdc.gov/ncbddd/hearingloss/ehdi-programs.html

37. NCHAM: State Grants. http://www.infanthearing.org/stategrants/2011-archive.php

38. 2001 CDC State EHDI Grant: North Carolina Abstract & Narrative. http://www.infanthearing.org/stategrants/cdc2001/cdc2001_northcarolina.html

39. Harrison W, Goodman D (2015): Epidemiologic trends in neonatal intensive care, 2007-2012. *JAMA Pediatr*. 169(9): 855–62.

40. Dabbous AO (2012): Characteristics of auditory brainstem response latencies in children with autism spectrum disorders. *Audiol Med*. 10(3): 122–31.

41. Miron O, Ari-Even Roth D, Gabis LV, Henkin Y, Shefer S, Dinstein I, *et al. (2015): Prolonged auditory brainstem responses in infants with autism. Autism Res*

42. Wong V, Wong SN (1991): Brainstem auditory evoked potential study in children with autistic disorder. *J Autism Dev Disord*. 21(3): 329–40.

43. Sersen EA, Heaney G, Clausen J, Belser R, Rainbow S (1990): Brainstem auditory-evoked responses with and without sedation in autism and down’s syndrome. *Biol Psychiatry*. 27(8): 834–40.

44. Ornitz EM, Mo A, Olson ST, Walter DO (1980): Influence of click sound pressure direction on brainstem responses in children. *Audiology*. 19(3): 245–54.

45. Gillberg C, Rosenhall U, Johansson E (1983): Auditory brainstem responses in childhood psychosis. *J Autism Dev Disord*. 13(2): 181–95.

46. Roth DA, Muchnik C, Shabtai E, Hildesheimer M, Henkin Y (2012): Evidence for atypical auditory brainstem responses in young children with suspected autism spectrum disorders. *Dev Med Child Neurol*. 54(1): 23–29.

47. Tas A, Yagiz R, Tas M, Esme M, Uzun C, Karasalihoglu AR (2007): Evaluation of hearing in children with autism by using teoae and abr. *Autism*. 11(1): 73–79.

48. Ververi A, Vargiami E, Papadopoulou V, Tryfonas D, Zafeiriou D (2015): Brainstem auditory evoked potentials in boys with autism: still searching for the hidden truth. *Iran J Child Neurol*. 9(2): 21–28.

49. Tanguay PE, Edwards RM (1982): Electrophysiological studies of autism: the whisper of the bang. *J Autism Dev Disord*. 12(2): 177–84.

50. Tharpe AM, Bess FH, Sladen DP, Schissel H, Couch S, Schery T (2006): Auditory characteristics of children with autism. *Ear Hear*. 27(4): 430–41.

51. Russo NM, Skoe E, Trommer B, Nicol T, Zecker S, Bradlow A, *et al. (2008): Deficient brainstem encoding of pitch in children with autism spectrum disorders. Clin Neurophysiol*. 119(8): 1720–31.

52. Rosenblum SM, Arick JR, Krug DA, Stubbs EG, Young NB, Pelson RO (1980): Auditory brainstem evoked responses in autistic children. *J Autism Dev Disord*. 10(2): 215–25.

53. Fujikawa-Brooks S, Isenberg AL, Osann K, Spence MA, Gage NM (2010): The effect of rate stress on the auditory brainstem response in autism: a preliminary report. *Int J Audiol*. 49(2): 129–40.

54. Magliaro FC, Scheuer CI, Assumpção Júnior FB, Matas CG (2010): Study of auditory evoked potentials in autism. *Pro Fono*. 22(1): 31–36.

55. Rumsey JM, Grimes AM, Pikus AM, Duara R, Ismond DR (1984): Auditory brainstem responses in pervasive developmental disorders. *Biol Psychiatry*. 19(10): 1403–18.

56. Grillon C, Courchesne E, Akshoomoff N (1989): Brainstem and middle latency auditory evoked potentials in autism and developmental language disorder. *J Autism Dev Disord*. 19(2): 255–69.
